# Making the Unseen Seen: The Role of Signaling and Novelty in Rating Metaphors

**DOI:** 10.1007/s10936-024-10076-7

**Published:** 2024-04-12

**Authors:** Kathleen Ahrens, Christian Burgers, Yin Zhong

**Affiliations:** 1 Department of English and Communication & Research Centre for Professional Communication in English, The Hong Kong Polytechnic University, Kowloon, Hong Kong; 2 Amsterdam School of Communication Research (ASCoR), University of Amsterdam, Amsterdam, The Netherlands; 3 Center for Language Education, The Hong Kong University of Science and Technology, Kowloon, Hong Kong

**Keywords:** Conventional Metaphor, Novel Metaphor, Metaphor Signal, Interpretability, Acceptability

## Abstract

Comprehension of metaphorical expressions differs with their degree of novelty. Conventional metaphors are typically comprehended as easily as literal sentences, while novel metaphors are responded to less quickly than their conventional counterparts. However, the influence of metaphor signals on the interpretability and acceptability of sentences with metaphors, especially their potential interaction with novelty, remains an open question. We conducted six online experiments among 1,694 native speakers of American English to examine how interpretability and acceptability ratings of individually presented sentences were affected by metaphor novelty and different types of metaphor signals. Across all six experiments, we consistently found that novel metaphors decreased the interpretability and acceptability of sentences compared to both conventional metaphors and literal controls. Signals, on the contrary, did not impact the interpretability or acceptability of the sentences. Moreover, only in experiment 3b did we find an interaction between metaphor type and signals. Specifically, when a metaphor was marked by double signals (i.e., both lexical signals and a typographical signal were added around the metaphorical keywords) vs. no signals, acceptability of novel metaphors increased, but acceptability of conventional metaphors decreased. We hypothesize that the double signaling of novel metaphors marks their novelty, making them more acceptable. By contrast, the double signaling of conventional metaphors may have been perceived as redundant, leading to a lower acceptability.

## Introduction

Metaphors involve cross-domain mappings between source and target domains (Gibbs, 1994; Lakoff & Johnson, 1980, 1999). For instance, the word “path” in the sentence “Many graduates take time to find their *path* after graduation” discusses the target domain of life in terms of the source domain of journey. Metaphorical expressions vary in the degree to which they are conventionalized. Highly conventionalized metaphorical expressions (i.e., conventional metaphors), such as “path”, are commonly used in everyday discourse. In fact, speakers and hearers may not even recognize “path” as figurative during the course of their conversation. Highly novel metaphoric expressions, by contrast, sound more unusual. For example, consider “Many graduates take time to find their *flavor* after graduation.” In this sentence, “flavor” is used creatively, presumably to attract the listener’s attention (e.g., Steen, 2008; Steen, 2011).

Previous cognitive and psycholinguistic studies found that the degree of novelty of metaphorical expressions influenced comprehension, with conventional (vs. novel) metaphors being processed more quickly and being perceived as more interpretable and acceptable (e.g., Ahrens, 2010; Ahrens & Gong, 2021; Bowdle & Gentner, 2005). Novel metaphors are therefore processed differently from conventional expressions in both cognition and communication (e.g., Gibbs & Tendahl, 2006; Steen, 2008, 2018; Tendahl & Gibbs, 2008).

Signaling is another device that could impact the perception of metaphors in cognition and communication. Signals (Goatly, 2011 [1997]; Skorczynska & Ahrens, 2015) or tuning devices (Cameron & Deignan, 2003) refer to discourse markers (e.g., particles, words, and phrases) that frequently occur with metaphors in discourse, such as modals (e.g., *must, would*), intensifiers (e.g., *actually*, *literally*), conditionals (e.g., *imagine*, *as it were*), metaphor flags^1^ (*e.g., like, as if*), and explicit markers (e.g., *metaphorically speaking*). Some lexical signals may be used to draw attention to the fact that a metaphor is being used, as in the case of the explicit metaphor flag “like” in the sentence, “She swims *like* a fish.” By contrast, other lexical signals can weaken the tone of a metaphor (Semino, 2008, p. 28) or play an essential role in guiding the reader or listener in their interpretation of the subsequent metaphorical expressions. For example, “kind of” or “sort of,” as seen in the sentence, “She is *sort of* a human encyclopedia when it comes to history.”

In addition to lexical signals, typographic signals such as scare quotes (“ ”) do not only potentially mark metaphors (Goatly, 2011 [1997]; Pasma, 2011), but can also signal the possibility of irony or doubt (Burgers & Steen, 2017; Ahrens, 2023) and thereby add another level of rhetorical complexity to the metaphor. For instance, consider the sentence, “He was ‘over the moon’ with his test results,” the use of scare quote highlights the metaphorical interpretation of the phrase, and could potentially signal irony or doubt depending on the context—if the test was known to be easy. Furthermore, scare quotes can be used to highlight an uncertain lexical choice that at the same time guides the reader toward a metaphorical interpretation (Nacey, 2013). Therefore, the presence of typographic signals, such as scare quotes, occurring on metaphorical expressions may increase an audience’s awareness of metaphor usage.

Previous research demonstrated that metaphor novelty and lexical signals can work as two independent processes as signals could occur when no metaphors were present or could also be used both with conventional and novel metaphors (Cameron & Deignan, 2003; Nacey, 2013). In an experimental study, Krennmayr et al. (2014) investigated the effect of lexical signals (using similes in particular) on perceived metaphor conventionality. Their results showed that readers of a business news text containing underlying racing metaphors (e.g., *accelerating* economy, *stalled* economy, economy could *veer off course*) were likelier to build their textual representation of the article on a metaphorical schema when the racing expressions were novel (vs. conventional) and when the mapping was lexically signaled (vs. unsignaled; e.g., “Economic development is a challenging and competitive process, *very much like* auto racing”). Krennmayr et al. (2014) further suggested that metaphors, particularly conventional ones, tended to go unnoticed without such a signal. In contrast, Gibbs (2015) did not find an effect for lexical signals to enhance people’s interpretation of cross-domain mappings of conventional metaphors. Burgers et al. (2012, Experiment 2), however, demonstrated that, for the rhetorical figure of irony, such signals could reduce complexity and increase comprehension.

Thus, previous results have demonstrated that both the degree of the novelty of metaphorical expressions (e.g., Bowdle & Gentner, 2005) and signaling (e.g., Krennmayr et al., 2014) have a role in alerting people’s awareness to metaphorical uses, with the findings on novelty being more uniform than those on signaling (cf. Gibbs, 2015). In addition, previous work on signaling has not looked at typographical signals, such as scare quotes, which provide visual information that may focus attention on the metaphorical usage, either alone or in combination with the lexical signals. Moreover, a potential interaction between the two factors (i.e., degree of novelty and metaphor signals) in the comprehension of metaphors has yet to be established.

This study aims to fill the gap on the effect of signaling on metaphors, especially to what degree novel metaphors, signaled metaphors, and novel and signaled metaphors impact the interpretability and acceptability of target sentences. Interpretability judgments ask participants to judge how easy or hard a sentence is to understand (Gibson & Fedorenko, 2013). By contrast, acceptability judgments are considered to primarily involve grammaticality judgements as they ask participants how acceptable or unacceptable as sentence is (see Schütze (1996/2016) who argues that “fully grammatical sentences can be judged as such without much reference to their meaningfulness” (p. 70).

First, we argue that novelty will decrease interpretability and acceptability, in line with previous findings (Ahrens, 2010; Ahrens & Gong, 2021; Bowdle & Gentner, 2005):

*H1: Sentences with novel metaphors (vs. conventional and literal sentences) are perceived as (a) less interpretable and (b) less acceptable.*

Second, we propose that signals can attenuate these effects, in that they may reduce the complexity of novel metaphors, making them more interpretable and acceptable. By contrast, we do not expect such an effect for conventional metaphors or literal statements, as these may already be perceived as interpretable and acceptable *without* such signals. This leads to:

*H2: For novel metaphors, signals (vs. no signals) increase (a) the interpretability and (b) the acceptability of target sentences. By contrast, for conventional metaphors and literal statements, signals (vs. no signals) do not affect either (c) the interpretability or (d) the acceptability of target sentences.*

## Methods

### Study Design

We conducted a series of six experiments; three studies asked for interpretability judgments (Experiments 1a, 2a, and 3a), and another three asked for acceptability judgments (Experiments 1b, 2b, and 3b). Each experiment had a 3 (novel metaphor, conventional metaphor, literal) x 2 (signal present vs. absent) mixed design. Each participant saw only one experimental sentence from each of the six stimulus sets. We also added four filler items across all experiments to serve as attention checks. That means, each participant reads ten sentences in total, including one sentence from each of the conditions of (1) unsignaled conventional metaphor, (2) unsignaled novel metaphor, (3) unsignaled literal control, (4) signaled conventional metaphor, (5) signaled novel, (6) signaled literal control, as well as four filler sentences. We randomized the stimuli to ensure that each participant would see each condition no more than once, and each participant would see no more than one condition from each stimulus set.

Our experiments differed from each other in two important ways. First, we varied the **type of signal** we used in each experiment. In Experiment 1, participants saw a sentence with one **lexical signal** (e.g., *During her last year in college, she consideredpossiblepaths for her future after graduation*).^2^ In Experiment 2, target words (i.e., metaphors) were marked with the **typographic signal** of scare quotes (e.g., *During her last year in college, she considered “paths” for her future after graduation).* In Experiment 3, we combined the lexical and typographic signals from Experiments 1 and 2, and participants saw a sentence with double signals (e.g., *During her last year in college, she consideredpossible“paths” for her future after graduation)* as compared to sentences with neither lexical nor typographical signals.^3^

The second difference was in the dependent variable we measured. In the experiments labeled with “a” (i.e., Experiments 1a, 2a, and 3a), we measured the perceived *interpretability* of each sentence, with participants evaluating how hard or easy it was to understand on a 7-point Likert scale, from 1 = *very hard to understand* to 7 = *very easy to understand* = 7. In the experiments labeled with “b” (i.e., Experiments 1b, 2b, and 3b), we measured the perceived *acceptability*, with participants deciding how unacceptable or acceptable they found each sentence, from 1 = *very unacceptable* to 7 = *very acceptable* = 7.

### Stimuli

Metaphor stimuli were generated from several reference dictionaries, including *Collins Cobuild English Guide: Metaphor* (Deignan, 1995), *Macmillan Dictionary* (https://www.macmillandictionary.com/), *Longman Dictionary of Contemporary English* (https://www.ldoceonline.com/), and SUMO (Suggested Upper Merged Ontology, http://ontology.teknowledge.com). The stimuli were then selected from several basic source domains, including journey, food, building, disease, sport, war, food, plant, product, and weather; whereas the target domains included life, idea, poverty, and relationships.

To create comparable pairs of conventional and novel metaphors, we ensured that stimuli of both conventional metaphors and novel metaphors were mapped onto the same target domain. In addition, a closely matched literal control sentence was created for each pair of conventional and novel metaphors. A sample of the six types of sentences included in the experimental materials is shown in Table 1.

**Table 1:** Examples of the six types of sentences included in the experimental materials (Experiment 1)

	Conventional Metaphor	Novel Metaphor	Literal Control
Unsignaled	During her last year in college, she considered **paths** for her future after graduation.	During her last year in college, she considered **flavors** for her future after graduation.	During her last year in college, she considered **scenarios** for her future after graduation.
Signaled	During her last year in college, she considered *possible ***paths** for her future after graduation.	During her last year in college, she considered *possible ***flavors** for her future after graduation.	During her last year in college, she considered *possible ***scenarios** for her future after graduation.

*Note.* The bolded words in the sentences are the words that varied for each condition type. The italicized words in italics are the signals. Note that signals and metaphor-related words were not marked in the experiments

To ensure that the novel metaphors are novel in the sense that they are rarely (if ever) used in daily expressions, we measured the frequencies of the target expressions in the three experimental conditions in a large-scale corpus. The occurrence of novel metaphor is significantly less frequent (14 instances) compared to their conventional (5904 instances) and literal (1770 instances) counterparts. See Digital Appendix A for details (https://osf.io/cdwp9/).

Possible lexical signals for metaphors were collected from previous literature (Skorczynska & Ahrens, 2015, Goatly, 2011 [1997]; Krennmayr, 2011; Nacey, 2013; Pasma, 2011). We selected signals that collocated in a stylistically natural way with the keyword across all three conditions using two modals (*possible, certainly*), four intensifiers (*just, literally, regular, actually*), one perceptual process (*viewed as*), and one superordinate term (*kind of*). In addition, the typographical signal of scare quotes occurring around the keyword was also selected. This type of signal allows for a variety of permutations to be explored in terms of how much information is highlighted for metaphors (i.e., only lexical signals (Experiment 1), only typographical signals (Experiment 2), or lexical and typographical signals together (Experiment 3), providing an opportunity to see if different types of signals have different effects on acceptability and interpretability ratings of conventional and novel metaphors.

Each set of sentences had a conventional metaphor condition, a novel metaphor condition, and a literal control condition occurring both with and without signals. The full set of experimental sentences included six sentence types and 36 sentences in total and can be found in Digital Appendices B, C, and D (https://osf.io/cdwp9/).

### Data Collection

Data were collected through SurveyMonkey (www.surveymonkey.com). Participants were recruited through Amazon’s Mechanical Turk (http://www.mturk.com). We limited participants’ location to the United States and their approval rate on MTurk to above 95%. In exchange for participation, each participant was paid US$0.80.

We invited 300 participants per experiment initially to obtain at least 240 unique workers for each experiment in this study. Three exclusion criteria were decided upon prior to running the study. Participants were excluded if at least one of three conditions was met: (1) responses showing the highest education level as below college, (2) responses showing English was not the only language they grew up speaking, and (3) the standard deviation (SD) of the four filler sentences in all the remaining responses was below 1.00.^4^

### Participants

All 1,694 participants were native English speakers with a partial college education or higher. Additional demographic information of the participants in the six experiments is shown in Table 2.

**Table 2:** Demographic information of participants in the six experiments

Experiment	Gender		M_Age_	SD_Age_
1a (lexical signal interpretability) (*N* = 267)	Female	*n* = 117 (43.8%)	35.94	11.75
	Male	*n* = 150 (56.2%)		
1b (lexical signal acceptability) (*N* = 279)	Female	*n* = 98 (35.1%)	37.47	11.14
	Male	*n* = 181 (64.9%)		
2a (typographic signal interpretability) (*N* = 283)	Female	*n* = 125 (44.2%)	39.13	11.98
	Male	*n* = 155 (54.8%)		
	Other	*n* = 3 (1.06%)		
2b (typographic signal acceptability) (*N* = 278)	Female	*n* = 126 (45.3%)	37.87	10.74
	Male	*n* = 152 (54.7%)		
3a (double signal interpretability) (*N* = 278)	Female	*n* = 137 (49.3%)	40.69	12.22
	Male	*n* = 141 (50.7%)		
3b (double signal acceptability) (*N* = 309)	Female	*n* = 159 (51.5%)	42.57	12.40
	Male	*n* = 150 (48.5%)		

### Data Analysis

Data were analyzed using *R* (R Core Team, 2021). The R packages *lme4* (Bates et al., 2015) and *sjPlot* (Lüdecke, 2021) were used to fit linear mixed-effects models. Fixed independent variables were type of utterance (i.e., conventional metaphor, novel metaphor, and literal control) and signaling (signals present vs. absent). Dependent variables included perceived interpretability or acceptability ratings. As random effects, intercepts for by-participants and by-stimulus were first included in the model; after that, we added the predictor of both the metaphorical conditions and signaled conditions. The interaction between metaphorical conditions and signaled conditions was also included as the predictor for the perceived interpretability and acceptability ratings. For the *post-hoc* comparisons, Kenward-Roger’s method was used to estimate degrees of freedom (Kenward & Roger, 1997), and Tukey’s *p*-adjustment correction method was adopted to compare across the conditions. Data and R codes of the analyses reported in this paper are publicly accessible at https://osf.io/cdwp9/.

## Results

### Experiments 1a and 1b: Lexical Signals Only

**Fig. 1:**
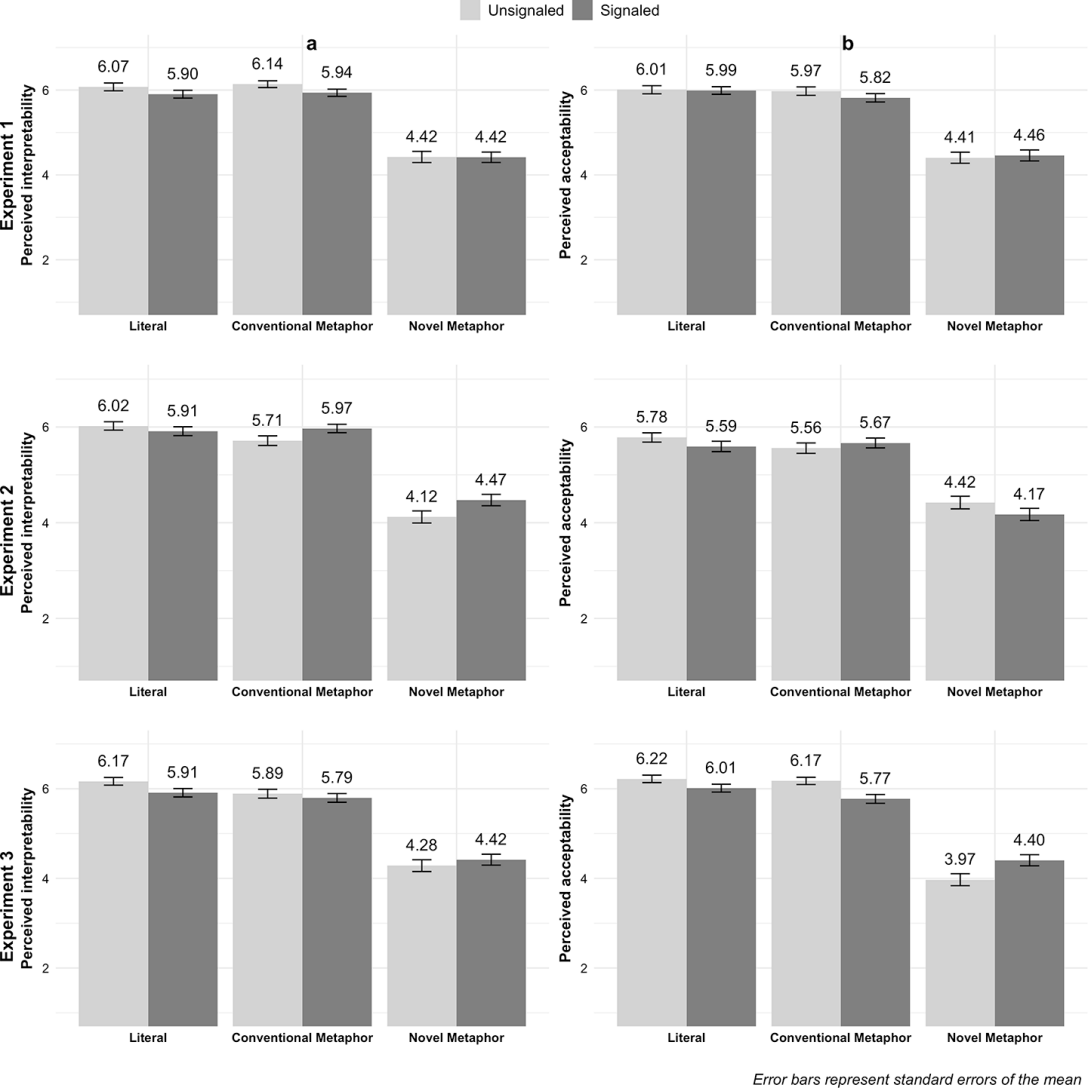
Means (and standard errors) of perceived interpretability and acceptability by type of utterance (literal control, conventional metaphor, novel metaphor) and signaling (unsignaled vs. signaled)

**Fig. 2:**
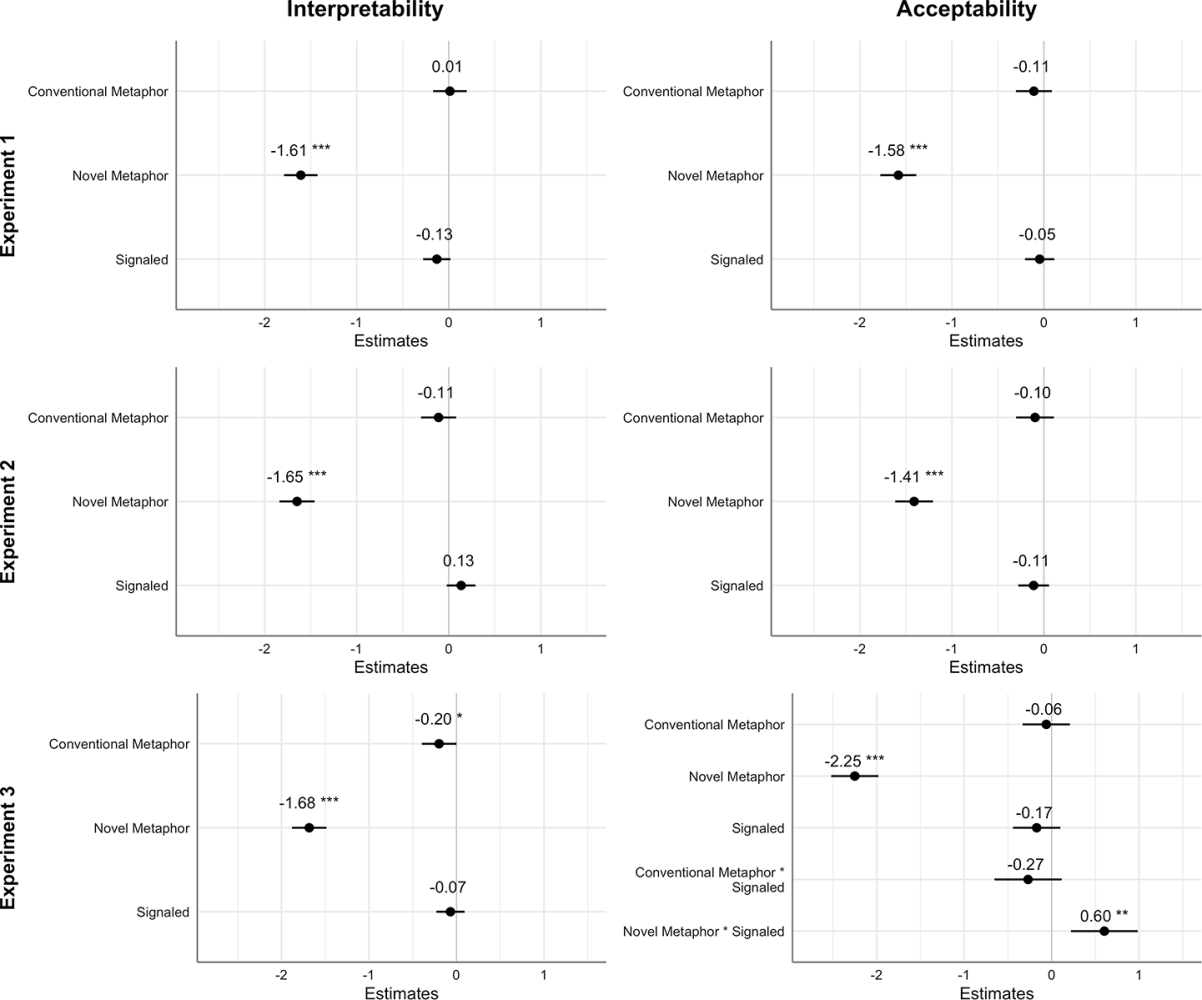
Effects of type of utterance and signaling on interpretability and acceptability. *Note:* Bars indicate 95% Confidence Intervals. For Type of Utterance, the Literal Control was the comparison condition; for Signaling, the Unsignaled Sentence was the comparison condition. * *p* < .05, ** *p* < .01, *** *p* < .001

Figure 1 shows descriptive statistics and Figure 2 contains model results (see Digital Appendices E and F for results in numeric format, https://osf.io/cdwp9/). In Experiments 1a and 1b, the signaling condition contained lexical signals only. First, we found no main effects of signaling on interpretability (*b* = -0.13, *95%CI* [-0.28, 0.02]), *p* = .09, β = -0.07, standardized 95% CI[-0.15, 0.01]) and acceptability (*b* = -0.05, *95%CI* [-0.21, 0.11]), *p* = .57, β = -0.02, standardized 95% CI[-0.11, 0.06]).

*H1* predicted that sentences with novel metaphors (vs. conventional and literal sentences) would be perceived as (a) less interpretable and (b) less acceptable. Results supported *H1a*, in that, compared to novel metaphors, both literal controls and conventional metaphors were seen as more interpretable (literal control: *b* = 1.61, *95%CI* [1,43, 1,79], SE = 0.09, *t*(1333) = 17.26, *p* < .0001, β = 0.88, standardized 95% CI[0.78, 0.98]; conventional metaphor: *b* = 1.62, SE = 0.09, *t*(1333) = 17.40, *p* < .0001, β = 0.88, standardized 95% CI [0.78, 0.98]), and acceptable (literal control: *b* = 1.58, *95%CI* [1.39, 1.78], SE = 0.10, *t*(1393) = 15.84, *p* < .0001, β = 0.81, standardized 95% CI[0.71, 0.91]; conventional metaphor: *b* = 1.47, SE = 0.10, *t*(1393) = 14.75, *p* < .0001, β = 0.76, standardized 95% CI [0.66, 0.86]). Conventional metaphors, on the contrary, did not differ from the literal controls in interpretability (*b* = 0.01, *95%CI* [-0.17, 0.20], *SE* = 0.09, *t*(1333) = 0.14, *p* = .89, β = 0.01, standardized 95% CI[-0.09, 0.11]) and acceptability (*b* = -0.11, *95%CI* [-0.30, 0.09], *SE* = 0.10, *t*(1393) = 1.09, *p* = .28, β = -0.06, standardized 95% CI[-0.16, 0.04]).

*H2* predicted an interaction effect between type of utterance and signaling. For almost all experiments, likelihood ratio tests showed that perceived interpretability and acceptability was best explained when only the main effects (and no interaction effect) of the metaphorical and signaled conditions were included in the model. This means that *H2* was not supported in these experiments.

The only exception was Experiment 3b, in which the model with main effects and the interaction between the metaphorical and signaled conditions had the best fit. Note that the reported results in Fig. 2 only showed results from the best-fitted models for each experiment.

### Experiments 2a and 2b: Typographic Signals Only

In Experiments 2a and 2b, the signaling condition contained scare quotes placed around the keywords. Again, we found no main effect for signaling on interpretability (*b* = 0.13, *95%CI* [-0.02, 0.29]), *p* = .09, β = 0.07, standardized 95% CI[-0.01, 0.15]) and acceptability (*b* = -0.11, *95%CI* [-0.28, 0.06]), *p* = .19, β = -0.06, standardized 95% CI[-0.14, 0.03]).

Results were similar to Experiment 1: compared to novel metaphors, the literal controls and conventional metaphors were rated as more interpretable (literal control: *b* = 1.65, *95%CI* [1.46, 1.84], *SE* = 0.10, *t*(1413) = 16.87, *p* < .0001, β = 0.87, standardized 95% CI [0.77, 0.97]); conventional metaphor: *b* = 1.54, *95%CI* [0.71, 0.91], *SE* = 0.10, *t*(1413) = 15.74, *p* < .0001, β = 0.81) and acceptable (literal control: *b* = 1.41, *95%CI* [1.20, 1.62], *SE* = 0.11, *t*(1388) = 13.43, *p* < .0001, β = 0.71, standardized 95% CI [0.60, 0.81]; conventional metaphor: *b* = 1.31, *SE* = 0.11, *t*(1388) = 12.52, *p* < .0001, β = 0.66, standardized 95% CI [0.56, 0.76]), supporting *H1*. We found no differences between conventional metaphors and literal controls in either interpretability (*b* = -0.11, *95%CI* [-0.30, 0.08], *SE* = 0.10, *t*(1413) = 1.31, *p* = .26, β = -0.06, standardized 95% CI [-0.16, 0.04]) or acceptability (*b* = -0.10, *95%CI* [-0.30, 0.11], *SE* = 0.11, *t*(1388) = 0.91, *p* = .36, β = -0.05, standardized 95% CI [-0.15, 0.05]).

Like in Experiment 1, the models without interaction terms had the best data fit, which means that *H2* was not supported.

### Experiments 3a and 3b: Lexical and Typographic Signals

In Experiments 3a and 3b, the signaling condition contained both lexical signals and scare quotes placed around the keywords. Results mostly replicated those of Experiments 1 and 2. Signaling did not affect either interpretability (*b* = -0.07, *95%CI* [-0.23, 0.09]), *p* = .40, β = -0.04, standardized 95% CI [-0.12, 0.05]) or acceptability (*b* = -0.17, *95%CI* [-0.44, 0.10]), *p* = .22, β = -0.08, standardized 95% CI [-0.22, 0.05]).

We again found that, compared to novel metaphors, both literal controls and conventional metaphors were evaluated as more interpretable (literal controls: *b* = 1.68, *95%CI* [1.49, 1.88], *SE* = 0.10, *t*(1388) = 16.66, *p* < .0001, β = 0.87, standardized 95% CI [0.77, 0.98]; conventional metaphors: *b* = 1.48, *SE* = 0.10, *t*(1388) = 14.69, *p* < .0001, β = 0.77, standardized 95% CI [0.67, 0.87]) and acceptable (literal controls: *b* = 2.25, *95%CI* [1.98, 2.52], *SE* = 0.10, *t*(1546) = 19.97, *p* < .0001, β = 1.11, standardized 95% CI [0.98, 1.25]; conventional metaphors: *b* = 1.75, *95%CI* [0.77, 0.96], *SE* = 0.10, *t*(1546) = 17.96, *p* < .0001, β = 0.87), supporting *H1.* We also found no differences between conventional metaphors and literal controls in acceptability (*b* = -0.03, *95%CI* [-0.33, 0.21]), *SE* = 0.10, *t*(1546) = 2.01, *p* = .66, β = -0.03, standardized 95% CI [-0.16, 0.10]). However, in contrast to Experiments 1 and 2, we did find that conventional metaphors were slightly less interpretable than literal controls (*b* = -0.10, *95%CI* [-0.40, -0.00]), *p* = .049, β = -0.10, standardized 95% CI [-0.21, -0.00]).

For interpretability, the model without interaction terms fit the data best, indicating that *H2* was not supported. By contrast, for acceptability, the model with an interaction between type of utterance and signaling had the best fit. The model revealed an interaction between novel metaphors and signaling (*b* = 0.60, *95%CI* [0.22, 0.98], *p* = .002, β = 0.30, standardized 95% CI [0.11, 0.49]). Pairwise comparisons revealed that novel signaled metaphors were perceived as more acceptable than novel unsignaled metaphors (*b* = 0.43, *SE* = 0.14, *t*(1547) = 3.12, *p* = .002), which supports *H2b*. By contrast, conventional signaled metaphors were perceived as less acceptable than conventional unsignaled metaphors (*b* = -0.44, *SE* = 0.14, *t*(1547) = 3.18, *p* = .002), contradicting *H2d.* We found no difference between literal signaled and unsignaled metaphors (*b* = 0.17, *SE* = 0.14, *t*(1547) = 1.24, *p* = .216). Results for Experiment 3b suggest that double signals increased the perceived acceptability for novel metaphors, but reduced the perceived acceptability for conventional metaphors.

## Discussion and Conclusion

We conducted a series of experiments investigating the effects of novelty and signals on perceived interpretability and acceptability.

*H1* predicted that novel metaphors would be perceived as less interpretable and acceptable than conventional metaphors and literal controls. In all six experiments, results supported *H1*. These findings support previous research on the influence of novelty on acceptability and interpretability ratings of metaphors in English (Bowdle & Gentner, 2005) as well as in languages other than English (Ahrens, 2010). Thus, studies on metaphor in any field should first clarify if they are examining conventional metaphors or novel metaphors or both in their analysis. Additionally, when creating experimental stimuli for neurolinguistic or psycholinguistic experimental studies involving metaphor, degree of novelty should be carefully controlled for and/or measured.

*H2* predicted that signals (vs. no signals) would increase interpretability and acceptability of novel metaphors, but not of conventional metaphors or literal controls. Five out of six experiments did not support this hypothesis, in that we found no interaction between type of utterance and signaling. Thus, results from the first five experiments suggest that lexical or typographic signals per se do not make novel metaphors more interpretable and/or acceptable if either the lexical or the typographic signals are presented. In Experiment 3b, we did find an interaction between type of utterance and signaling, suggesting that double (vs. no) signals increased the acceptability of novel metaphors, but decreased the acceptability of conventional metaphors.

The contrast between acceptability and interpretability judgements is of interest for two reasons. First, acceptability judgements are viewed as judgements related to the relative grammaticality of a sentence, while interpretability judgements are related to a sentence’s meaning, including its “truth or plausibility in the real world”, which is considered orthogonal to questions of acceptability (Schütze, 2011). Given that the stimuli were made up of individual sentences (so as to tightly control for other variables), it suggests that participants were open to possible interpretations of the combined signals for conventional metaphors, but less open to accepting those sentences as grammatical.

Second, our results suggest that the use of the typographic and lexical signals together (when compared with no signals) had the effect of marking the target expressions as explicitly figurative, which was considered more acceptable for novel usages, but less acceptable for conventional usages. The inverse effects of double signaling on conventional and novel expressions in the acceptability condition may be explained by the potential rhetorical function of scare quotes as indicating irony or uncertainty. That is, since conventional metaphors are already well-established and accepted, the use of explicit double signaling becomes incongruous and redundant, which, in turn, may have negatively influenced acceptability ratings. For novel metaphors, however, such signaling is useful in that it highlights the novelty of the language used. That is, novel language use is acceptable, but marking conventional language use is not when there is no context for doing so.

Similar to previous findings (Krennmayr et al., 2014; Gibbs, 2015), signals in our study did not enhance the interpretation of sentences with metaphors, suggesting that participants were more open to possibilities of interpretation in sentential meaning irrespective of whether or not it was marked. Decisions regarding acceptability, however, were stricter, suggesting that future studies should select the appropriate judgement task accordingly (Gibson & Fedorenko, 2013). For example, if one were to set up a scenario in which a particular conceptual metaphor is used in a number of different times in coherent manner throughout the passage, one would expect that acceptability ratings for a target sentence that contained a typographically signaled conventional metaphor from the same source-target domain mapping to be less acceptable than a literal control condition because there is no reason for it to be marked in that context. However, if the target sentence contained a metaphor that was either highly novel from the same source-domain mapping or instead was from a completely different source-target domain mapping, then typographically signaling either type of metaphor (both of which are novel in this scenario but for different reasons) should be considered more acceptable compared to a control condition. Interpretability ratings, however, would not necessarily show the same set of distinctions, as a typographical signal would only draw attention to a range of possible meanings (including irony).

Across six experiments, our study revealed how different elements of metaphorical statements (novelty, presence of signals) impact perceptions of interpretability and acceptability. Overall, we found that novel metaphors are less interpretable and acceptable than conventional metaphors or literal controls. Signaling can increase the acceptability of novel metaphors, but only when signals are clearly visible (as in the case of double signals). By contrast, signaling can backfire in situations in which metaphorical statements are already interpretable and acceptable, as with conventional metaphors. In such situations, adding excessive signals may decrease interpretability and acceptability. These results demonstrate how different features of metaphors (novelty, signaling) can interact in influencing interpretability and acceptability.

## Footnotes

1 The term of “signal” is not used consistently in previous studies. For example, Gibbs (2015) named them “pragmatic signals,” while they were called “metaphor flags (MFlag)” in the Metaphor Identification Procedure VU University Amsterdam (MIPVU) (Steen et al., 2010).

2 The underlined word “possible” is a sample of metaphor signal. But note that we did not underline any signals when the stimuli were shown to the participants.

3 Again, all the signals were not underlined in the experiments.

4 This measurement is to exclude unreliable responses. The four filler sentences consisted of two highly interpretable and acceptable sentences and two uninterpretable and unacceptable sentences. The low SD of the four filler sentences (below 1.00) indicated a relatively low variation in a participant’s responses of the filler sentences (meaning they did not pay attention to the questions). When this occurred, we eliminated the participant from the dataset.
